# Molecular Characterization of Emerging *Gyrovirus galga* 1 from Poultry Markets of Guangxi, China

**DOI:** 10.3390/ijms27041674

**Published:** 2026-02-09

**Authors:** Yanfang Zhang, Zhixun Xie, Zhiqin Xie, Liji Xie, Meng Li, Ming Yan, Aiqiong Wu, Minxiu Zhang, Qing Fan, Tingting Zeng, Jiaoling Huang, Sheng Wang, Lijun Wan, Xiaofeng Li, You Wei, Sisi Luo

**Affiliations:** 1 Guangxi Key Laboratory of Veterinary Biotechnology, Guangxi Veterinary Research Institute, Nanning 530001, Chinazhminxiu2010@163.com (M.Z.);; 2 Key Laboratory of China (Guangxi)-ASEAN Cross-Border Animal Disease Prevention and Control, Ministry of Agriculture and Rural Affairs of China, Nanning 530001, China

**Keywords:** avian gyrovirus 2, *Gyrovirus galga* 1, epidemiological survey, molecular properties, poultry market

## Abstract

*Gyrovirus galga* 1 (GyG1) can infect a variety of animals and humans, but prevention and control strategies are limited, which endangers the healthy development of the poultry breeding industry and has a potential impact on public health safety. The live poultry market (LPM) connects the production and consumption ends, and the pathogen may spread across regions through transportation and personnel flow. To understand the prevalence of GyG1 in Guangxi, 3482 samples from LPMs, namely, 2693 chicken throat and cloacal swabs and 789 environmental samples collected in Guangxi from December 2019 to December 2024, were assayed by PCR. The results revealed that GyG1 was present in chicken and environmental samples from LPMs in Guangxi, China, with positivity rates of 17.08% and 13.31%, respectively. Eight GyG1-positive samples were randomly selected, including 5 chicken swab samples and 3 environmental samples for whole-genome amplification. The amino acids encoded by the three ORFs were analysed, and some mutation sites unique to these 8 variants were found. The homology between the 8 GyG1 genomes and 36 reference sequences was 96.8–99.8%. The homology of the VP1 gene sequence was 96.5–99.9%, and the homology of the amino acid sequence was 99.4–100%. A phylogenetic tree was constructed on the basis of the 8 GyG1 genomes and 36 GyG1 reference genome sequences from 14 different species (8 from zoos) in this study. The 44 sequences were divided into three branches constituting groups A, B and C, with the 8 novel strains classified into group A2. Recombination analysis predicted that two recombination events in the GyG1 sequence were associated with the emergence of Guangxi strain GX-AGV2-202109-5. This study clarified the prevalence and molecular characteristics of GyG1 in LPMs in Guangxi, China, were clarified for the first time, providing important data supporting the prevention and control of GyG1 infection and providing a reference for further understanding the epidemiology and genetic diversity of GyG1.

## 1. Introduction

*Gyrovirus galga* 1 (GyG1), formerly known as avian gyrovirus 2 (AGV2), a circular virus, was the first *Gyrovirus* discovered since the discovery of chicken infectious anaemia virus (CIAV) in 1979. GyG1 was initially characterized in 2008 when Rijsewijk et al. (2011) sequenced serum samples from diseased 3-week-old chickens on a farm in southern Brazil that exhibited weight loss and neurological impairment [1]. The amplified nucleotide sequence exhibited homology of up to 40% with CIAV and was therefore named avian gyrovirus 2 (AGV2). According to the 2022 International Committee on Taxonomy of Viruses (ICTV) (https://ictv.global/taxonomy) (accessed on 1 December 2022), the genus *Gyrovirus* is in the family Anelloviridae. The genus *Gyrovirus* contains ten species, one of which is GyG1. Therefore, avian gyrovirus 2 was renamed *Gyrovirus galga* 1 (GyG1) in 2021.

GyG1 is a nonenveloped, small, icosahedral virus with a negative-sense single-stranded DNA (ssDNA) genome of approximately 2.38 kb belonging to the genus *Gyrovirus* in the family Anelloviridae [2], containing three open reading frames (ORFs) in its genome. ORF1 and ORF2 partially overlap, and encode the nonstructural proteins VP2 and VP3, respectively. ORF3 encodes VP1, which is a structural protein known to have good antigenicity. The homologous sequence encoding the CIAV protein is similar to that of GyG1, and the VP3 protein of GyG1 is also structurally similar to the VP3 protein of CIAV. Studies have shown that VP3 can induce apoptosis in cancer cells [3,4].

Although GyG1 infection alone may cause limited or subclinical disease, coinfection with other avian viruses, even nonpathogenic ones, can lead to high morbidity and mortality in chicken flocks [5]; for example, mixed infection with attenuated strains of GyG1 and CIAV was reported to cause a fatality rate of 56.67%, accompanied by severe organ damage, immunosuppression, and anaemia, brain injury, impaired cognitive development, weight loss, bleeding, oedema, glandular stomach erosion, and facial and head swelling, as reported in a necropsy study of dead chickens [6]. Lima et al. [7] performed metagenomic sequencing of the intestinal contents of healthy chickens from Brazilian commercial farms and identified GyG1. Mixed infections greatly increase the difficulty of preventing and treating GyG1 infection. Yan et al. [8] reported that the probability of GyG1 infection in chickens with transmissible viral proventriculitis was very high, according to long-read sequencing, and this high probability may have an impact on the synergistic occurrence of proventriculitis with other viral infections in chickens, but further investigation is still needed to determine the effect of GyG1 proventriculitis in commercial chickens.

Surveillance studies have revealed a remarkably wide distribution and host range for GyG1. Since 2011, GyG1 has been detected globally, including in China, France, Italy, South Africa, the USA, Brazil, the Netherlands, and Japan and in diverse samples, including skin swabs from healthy humans; blood from immunocompromised patients (e.g., transplant recipients and AIDS patients); diarrhoeal faeces; chicken tissues (frozen brain, wings, and feather pulp samples, dating back to 1997); and even commercial vaccines [9,10,11,12,13,14,15]. Furthermore, GyG1 has been identified in a broad spectrum of animals beyond poultry, including cats, dogs, ferrets, snakes, and numerous zoo animals (e.g., hippopotamuses, tigers, lions, egrets, pheasants, peafowls, and sika deer), and ticks [11,16,17,18,19,20].

These findings indicate that GyG1 is widely distributed. The clinical symptoms of GyG1 infection are occult, and most infections occur as mixed infections, which increases the difficulty of preventing, controlling, and treating the disease. GyG1 poses not only poses an economic threat to the poultry industry but also carries potential risks to public safety. Therefore, investigating the epidemiology and molecular characteristics of GyG1 is necessary. To address the need for regional epidemiological data, this study aimed to determine the prevalence of GyG1 in chicken populations in Guangxi, China. In this study, we investigated the prevalence of GyG1 in chicken swab samples from different live poultry markets (LPMs) in Guangxi using a PCR assay. Eight positive samples were sequenced to obtain the whole-genome sequence. Molecular characterization and phylogenetic analysis of the eight sequences were performed, with comparison to 36 GenBank reference sequences. This work aimed to elucidate the genetic variation and evolutionary relationships of GyG1 strains, thereby offering data to guide GyG1 infection prevention and control efforts in Guangxi.

## 2. Results

### 2.1. Poultry Market Flow Survey Results

**Figure 1:**
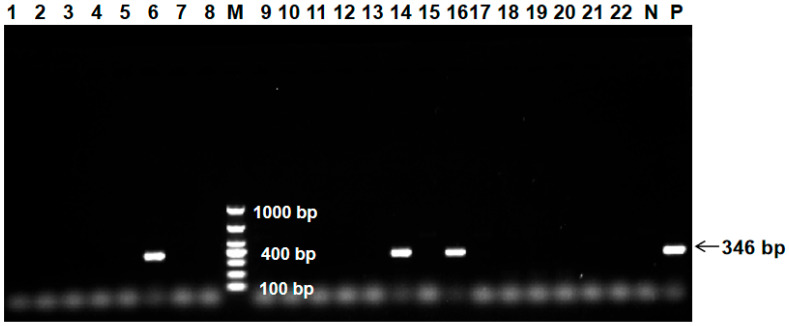
PCR detection results of some clinical samples. M: DL1000 DNA Marker; lanes 1–22: clinical samples; N: negative control; P: positive control; lanes 6, 14 and 16 showed positive PCR detection results, while all other numbered lanes show negative results.

To investigate the prevalence of GyG1 in LPMs across Guangxi, we first used PCR to determined the percentage of positive chicken swabs and environmental samples by PCR. We found that GyG1 was present in chickens and environmental samples collected from LPMs. The experiment included both negative and positive controls. The electrophoresis results for some clinical samples detected by PCR are shown in Figure 1. The results of sampling and testing in different years were detailed in Table 1. GyG1 was detected by PCR in 2693 chicken cotton swab samples and 789 environmental samples collected from different LPMs in Guangxi from December 2019 to December 2024, overall positivity rate was 16.23% (565/3482). The positivity rate for chicken swab samples was 17.08% (460/2693), while that for environmental samples was 13.31% (105/789). All environmental sample types tested positive, with positivity rates across different types decreasing in the following order: sewage, 28.57% (28/98); floors, 22.40% (28/125); chicken cages, 16.00% (20/125); bleeding buckets, 11.76% (10/85); machinery, 9.52% (8/84); chopping boards, 6.98% (6/86); kitchen knives, 3.53% (3/85); and tap water, 1.98% (2/101).

### 2.2. Overall Features of the Genomes

**Figure 2:**
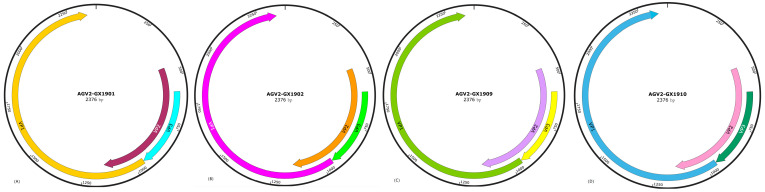

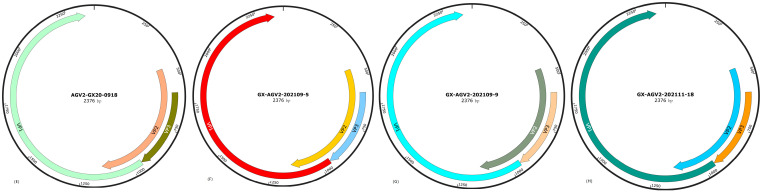
Genome organization of the 8 GyG1 strains isolated in Guangxi (**A**–**H**). The inset text indicates the virus names and genome sizes. ORFs (predicted using SnapGene Viewer) are depicted with internal arrows, where the location indicates the direction, the length represents the relative size, and the colour denotes the reading frame.

To quickly obtain a broad view of the mutations, virulence range and viral types of the GyG1 virus, we randomly selected 8 positive samples for whole-genome sequencing. Genome-wide analysis revealed that the lengths of the eight GyG1 genome sequences ranged from 2375 bp to 2376 bp. The whole-genome structures of the 8 GyG1 strains were essentially the same and were also similar to the genome structure of CIAV; all of the genomes were composed of ORF1, ORF2 and ORF3, encoding the VP2, VP3 and VP1 proteins, respectively (Figure 2A–H). Sequence alignment revealed no nucleotide insertions or deletions in the ORFs of the genes from the 8 GyG1 strains.

### 2.3. Nucleotide and Amino Acid Sequence Comparison

Clustal W (v2.0), DNAMAN (v10), and BioEdit (v7.0.9) were used to analyse the homology among the genome sequences of the 8 GyG1 strains. The results revealed that the homology among the 8 GyG1 strains was 99.4–99.9%, and their homology with the 36 reference sequences in GenBank was 90.5–99.9%. The homology of the 8 GyG1 amino acid sequences was 98.5–99.9%, and the amino acid homology with the 36 reference sequences was 87.3–100%. The sites of sequence variation within the 8 GyG1 whole-genome sequences compared with the reference sequence were concentrated mainly in the VP1 gene. For this reason, homology analysis of the GyG1 VP1 structural gene and amino acid sequences was further carried out. The results revealed that the homology of the 8 GyG1 VP1 gene sequences was 96.7–100%, and the homology with the 36 VP1 gene reference sequences was 93.1–100%. The homology of the deduced amino acid sequences of the eight GyG1 VP1 genes was 99.8–100%, and the homology with the 36 VP1 amino acid reference sequences was 97.2–100%.

**Figure 3:**
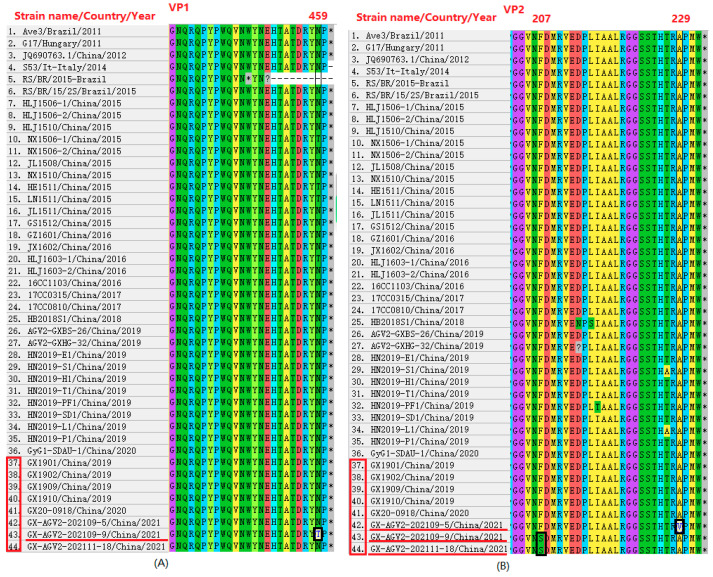
The amino acid substitutions in VP1 and VP2 are shown in (**A**,**B**). In the alignment chart, the red boxes correspond to the sequence of the strain investigated in this study. Mutated amino acid sites are indicated by black boxes, and strains exhibiting an amino acid variation are underlined in red.

Analysis of the eight sequences obtained in this study revealed that the amino acid homology among seven of the VP1 sequences was 100%. Only the VP1 amino acid sequence of the GyG1 isolate from the LPM environmental sample AGV2-202109-9 showed less than 100% homology—99.8%—with the other seven sequences. Further comparison with the 36 reference sequences demonstrated that the aforementioned seven VP1 sequences from this study exhibited 100% amino acid homology with the VP1 sequences of the reference strains S53/It, HLJ1506-1, HLJ1506-2, HLJ1510, NX1506-2, JL1508, NX1510, JX1602, and AGV2-GXBS-26. Among these reference strains, S53/It originated in Italy, while the others were all from China. AGV2-GXBS-26 was isolated from canine serum, and the remaining six reference sequences were isolated from chicken serum or faeces. Additionally, the VP1 sequence of AGV2-202109-9 showed 100% amino acid homology with the VP1 sequences of the reference strains NX1506-1, HE1511, LN1511, and HLJ1603-1. The 8 sequences had the lowest amino acid homology (97.2%) with the VP1 sequence of the RS/BR/2015 strain from Brazil. See Figure S1 in the Supporting Information for details. In this study, amino acid substitutions were identified in the VP1 and VP2 proteins of specific AGV2 strains obtained from LPM environment samples. In strain AGV2-202109-9, position 459 of VP1 showed a substitution of N to T, and position 207 of VP2 showed a substitution of F to S (Figure 3A,B). Furthermore, in strains AGV2-202109-5 and AGV2-202111-18, the VP2 protein exhibited an A to V substitution at position 229 and an F to S substitution at position 207 (Figure 3B). The corresponding nucleotide sequences are detailed in Figure S2 of the Supporting Information.

### 2.4. Phylogenetic Analysis

**Figure 4:**
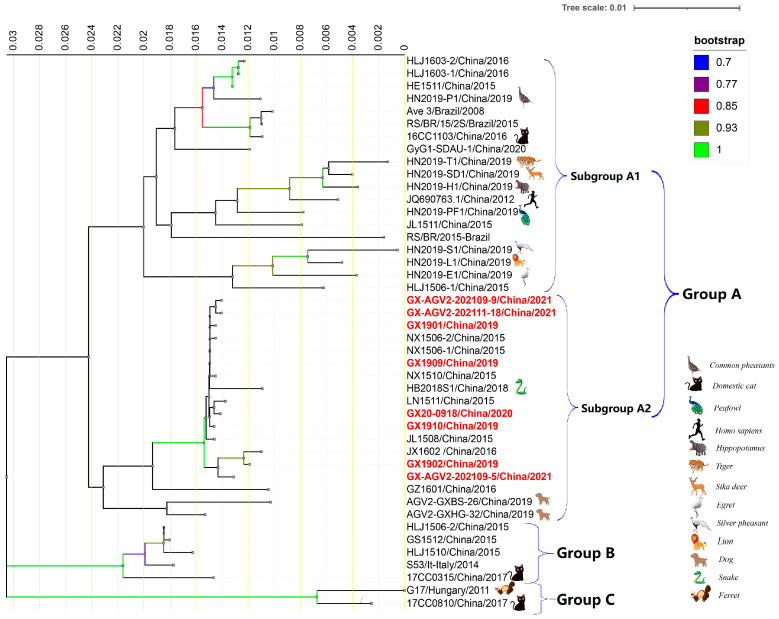
Phylogenetic trees were constructed using the nucleotide sequences of the full genomes of GyG1 strains. The tree were generated with 1000 bootstrap replicates. The bar scale indicates genetic distance, and bootstrap values are displayed at the nodes. Red font represents the Guangxi GyG1 strains.

In total, 36 reference sequences were downloaded from NCBI GenBank, and a phylogenetic tree of all the 44 GyG1 gene sequences was constructed using MEGA 11.0 software and ITOL (https://itol.embl.de/itol.cgi?tdsourcetag=s_pcqq_aiomsg) (accessed on 1 December 2024). Phylogenetic trees were constructed on the basis of the sequences of the VP1, VP2 and VP3 genes (Supporting Information Figure S3) and the whole-genome sequence (Figure 4). All the constructed phylogenetic trees revealed significant differences between the Guangxi GyG1 strains and the reference strains. In the trees constructed on the basis of the three genome segments and the whole genome, the Guangxi GyG1 strains are different from the other reference strains and are essentially in the same branch. A set of 44 GyG1 sequences obtained from 14 different species are shown in Figure 4 (the unmarked sequences in the figure are from chickens). GyG1 from common pheasants, peacocks, hippopotamuses, tigers, sika deer, egrets, silver pheasants, lions, humans, cats and 9 chicken sources from the China Zoo are in the A1 subgroup of group A in the same branch. The 8 Guangxi strains, 2 GyG1 strains from dogs, 1 GyG1 strain from snakes and 7 GyG1 strains from other chickens are closely related, and they are in the A2 subgroup of group A in the same branch. One GyG1 from a domestic cat and four GyG1 from chickens (including S53/It from Italy) are in the same branch, group B. One GyG1 from a Chinese cat and one GyG1 from a Hungarian ferret are in the same branch, group C.

### 2.5. Results of Recombination Analysis 

**Figure 5:**
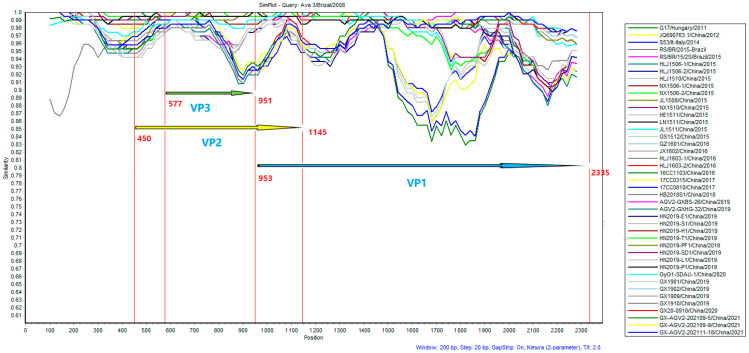
Full-length nucleotide sequences were analysed using SimPlot with Ave 3 as the query sequence. The line colours correspond to the 8 Guangxi GyG1 strains and the 36 reference GyG1 strains, with nucleotide positions referenced to the Ave3 sequence.

**Figure 6:**
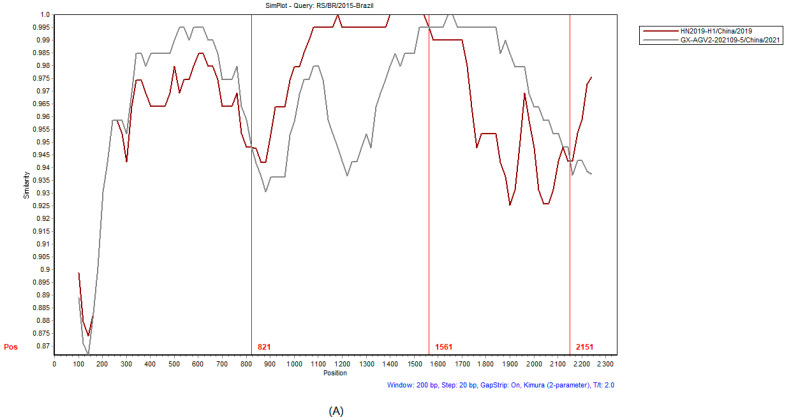

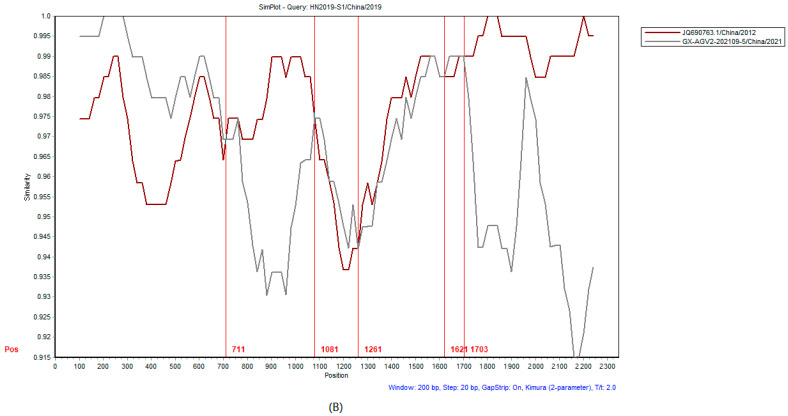
SimPlot analysis of the recombinant strains. The recombination events related to GX-AGV2-202109-5 (**A**,**B**).

Homology analysis of the 44 complete GyG1 genomic sequences conducted with SimPlot (Figure 5) revealed that the homology of the VP2 and VP3 genes was greater than that of the VP1 gene, demonstrating that the major variable region of GyG1 is located within the VP1 gene. Two recombination events in the GyG1 genome were predicted to be related to the Guangxi GX-AGV2-202109-5 strain (Table 2). The predicted recombinants were subsequently evaluated separately using SimPlot v3.5.1 software (Figure 6).

## 3. Discussion

In this study, a total of 3482 chicken swab and environmental samples collected from LPMs in Guangxi from December 2019 to December 2024 were analysed to understand the epidemiology of GyG1, and the overall positivity rate was 16.23%. Specifically, the positivity rate for the chicken swab samples was 17.08% (460/2693), while that for the environmental samples was 13.31% (105/789). The overall positivity rate was higher than that reported for large-scale chicken farms in Guangxi (9.96%) [21], reported the GyG1 positivity rate reported by Yao et al. [4] in other regions (12.48%, 55/448). However, it was lower than some of the positivity rates reported by Ye et al. [13] for four LPMs in other parts of China (12.5–25%), the rates reported by Zhang et al. [22] in chicken samples from various provinces in China (17.86–32.14%) and the positivity rate of symptomatic cases in northern Vietnam (20.63%) [23]. Moreover, it was substantially lower than the rates reported in the southern Netherlands (60.32%) and Brazil (60.4–90.7%) [24]. These findings indicate that GyG1 is ubiquitous in LPMs and their environments in Guangxi. This higher prevalence may be attributed to the complex and diverse sources of poultry in these markets, facilitating viral spread across different types of poultry. In addition, the frequent trading activities, the absence of market closure, and the prolonged presence of unsold poultry often comprising chickens and waterfowl from various sources cohabiting the same space may further promote cross-species transmission of the virus.

The 8 GyG1 whole-genome sequences obtained from samples collected at different times and locations were highly similar, but the backgrounds were not identical. Notably, this study reports the first detection of GyG1 in environmental samples. Although the virus is likely shed from infected poultry within the market, its presence in the environment demonstrates considerable contamination and underscores the role of fomite transmission as a potential transmission route. These findings highlight the need for regular disinfection and improved sanitary practices in LPMs to help interrupt viral transmission. Encouragingly, the positivity rate of GyG1 is currently showing a significant decreasing trend (Table 1), which may be closely related to the increasing emphasis on biosafety. Chickens in LPMs are sourced from different parts of Guangxi, which means that the detected strains may be epidemic strains in southern China. Another possibility is that these strains may have spread from one place to another through an unknown mechanism during this period. In addition, studies have shown that GyG1 may also be introduced by vaccines [15]. Given that its potential identity as a poultry vaccine pollutant may also be one of the reasons for its widespread detection, future studies need to include vaccine monitoring data to further clarify this issue.

The amino acid substitutions VP1-459, VP2-229, and VP2-207 were identified in the AGV2-202109-5 and AGV2-202109-9 strains. VP1 serves as the sole structural protein of GyG1, whereas VP2 acts as its regulatory protein, which has often been linked to viral replication or antigenic determinants [6,21,24]. Notably, the phosphorylase activity of the VP2 protein in CIAV plays a crucial role in viral replication within cells and in cellular pathogenicity [25]. We therefore speculate that these mutations may influence the host range or cell tropism of the virus. Although the functions of the VP2 protein of GyG1 are hypothesized to be similar to those of CIAV VP2, this assumption requires validation through further functional studies. Additionally, the abovementioned substitutions could alter the replication efficiency, capsid stability, or antigenic characteristics of the virus, thereby potentially affecting its transmission efficiency or immune evasion. Elucidating the functional significance of these mutations is anticipated to be an important focus of subsequent research.

Representative strains were selected from the positive samples detected by epidemiological investigation for whole-genome sequencing. A gene-based phylogenetic tree of the 8 GyG1 strains from Guangxi and 36 reference strains was constructed and divided into three major branches corresponding to groups A, B and C. The 8 GyG1 strains from Guangxi were closely related to 7 chicken-derived GyG1 strains, 1 snake-derived GyG1 strain and 2 dog-derived GyG1 strains from China. The 8 strains were classified into the A2 subgroup of group A, indicating that they may have common parental strains. Typically, viruses from the same country or geographical area tend to cluster together; the clustering pattern of the Guangxi strains in this study did not show obvious indications of introduced variation.

Recombination analysis revealed that the recombination events in the two early reference strains were related to the same isolate, GX-AGV2-202109-5, detected in this study, which was highly important. This finding suggests that these reference strains are not direct parents but share an unsampled common viral ancestor with our isolate GX-AGV2-202109-5. The ancestor acquired a gene fragment with significant adaptive advantages through recombination, which enabled the viral lineage containing this fragment to successfully spread and continuously evolve, ultimately being detected at different times and locations. These findings underscore the idea that sequencing time is not equivalent to the time of evolutionary origin and highlight the importance of strengthening virus monitoring to fill gaps in evolutionary history.

To date, GyG1 has been detected in 14 different hosts, but most studies still indicate that chickens are the main hosts. The specific timing and mechanism of viral spread of the virus among individuals of the same or different species are still unclear. To clarify the transmission pattern (single vs. multiple introductions) of the virus into Guangxi, genomic sequence analysis should be performed on strains from diverse locations. Notably, the results of the phylogenetic analysis revealed discrepancies among the trees constructed from the individual VP1, VP2, and VP3 genes (Supporting Information Figure S3) and the whole-genome tree (Figure 4). Consistent with the findings of GTH Tran et al. [23], this study indicates that nucleotide variation occurs predominantly in the VP1 gene. Therefore, phylogenetic analysis based on the VP1 gene alone is sufficient to represent whole-genome phylogeny. At present, studies on the geographical distribution, genetic diversity, cross-species infection potential and zoonotic risk of GyG1 are still relatively rare, and related explorations are ongoing.

This study has several limitations. First, no internal reference gene was used during DNA amplification, which could influence the accuracy of the quantification, despite the implementation of rigorous positive and negative controls and DNA quality assessments to ensure experimental reliability. Second, the sequenced samples were selected at random, and most were collected approximately five years before the analysis was conducted. While this approach facilitates the reconstruction of historical transmission lineages, these samples may not fully capture recently circulating strains, thereby limiting insights into current transmission dynamics. Future studies should consider employing exogenous synthetic internal controls and incorporating systematic longitudinal sampling over time periods and across geographic regions to more accurately elucidate spatiotemporal spread and evolutionary patterns.

## 4. Materials and Methods

### 4.1. Sample Collection

**Figure 7:**
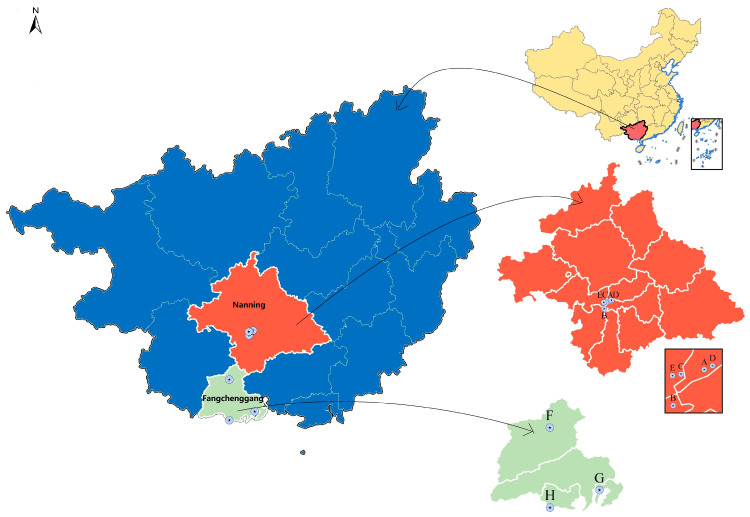
Geographical distribution of the sampling sites in live poultry markets. These markets comprised five markets in Nanning (A–E) and three in Fangchenggang (F–H).

From December 2019 to December 2024, a cluster sampling method was used to conduct an epidemiological survey of LPMs in Guangxi. A total of 3482 samples, namely, 2693 throat and cloacal swabs from chickens and 789 environmental swabs, were randomly collected from chickens and their environments in 8 LPMs in Nanning and Fangchenggang of Guangxi, China. Sampling details are provided in Table 1. Both studies were conducted in densely populated areas. The distance between the five markets in Nanning ranges from 1.9 to 12.5 km, whereas the three markets in Fangchenggang are 48 to 113 km apart (Figure 7). In accordance with the World Organization for Animal Health (OIE) protocol, the swab samples were placed in a centrifuge tubes containing 1 mL of PBS (containing 2000 U/mL penicillin and 2 mg/mL streptomycin), transported at 2–8 °C, and then stored at −80 °C until processing.

### 4.2. DNA Extraction, PCR Detection and Complete Genome Sequencing

Viral DNA was extracted from samples using a prepackaged viral RNA/DNA extraction kit (Biovet Biotechnology, Tianjin, China). The quality and concentration of the DNA were precisely evaluated after extraction, and the purity of the DNA was detected by NanoDrop 2000 (Thermo Fisher Scientific, Wilmington, DE, USA) spectrophotometer. After screening and comparison, we finally determined the sequences of the detection primers for GyG1. The presence of GyG1 in swab samples was detected by PCR [13], and a 346-bp fragment targeted to regions of the genome encoding the VP2 and VP3 genes was amplified (Supporting Information Table S1). Premix Taq ™ (Ex Taq ™ Version 2.0 plus dye) (Takara, Dalian, China) was used for PCR. All DNA extraction and PCR assays were performed with a positive control (Accession No. MW404234) and a negative control (sterile water).

Eight GyG1 positive samples were randomly selected for sequencing. These samples originated from six different markets, with three samples from the same market collected from different stalls at different time points. Twelve overlapping primers were designed to amplify the whole genome of the virus by referring to the primers used to amplify the complete GyG1 genome [3] to ensure the integrity of the results (Supporting Information Table S2). PCR amplification (Tks Gflex TM DNA Polymerase from Takara, Dalian, China) was performed using primers listed in Table S2 of the Supporting Information, and the annealing temperature and primer extension time were adjusted according to the length of the amplified product. Purified PCR amplicons were ligated into the TOPO Vector (Aidlab Biotech, Beijing, China) and subsequently sent to Sangon Biotech (Sangon Biotech, Shanghai, China) for Sanger sequencing. The obtained chromatograms were assembled for analysis.

### 4.3. Sequence Analysis

The 8 GyG1 whole-genome sequences obtained in this study were analysed, and spliced using the BioEdit (version 7.0; Tom Hall, Carlsbad, CA, USA). Sequence similarity was assessed by using DNAMAN (version 10; Lynnon Biosoft, San Ramon, CA, USA), while Clustal W (version 2.0; European Molecular Biology Laboratory—European Bioinformatics Institute (EMBL-EBI), Hinxton, Cambridgeshire, UK) was used to obtain the GyG1 whole genome sequence and sites of variation. All the ORFs were predicted using SnapGene Viewer (version 5.0.5; GSL Biotech LLC, Chicago, IL, USA). Comparative genomic analysis of the GyG1 strains was conducted via the mVISTA online platform, utilizing >36 aligned reference sequences obtained in different from regions and years. Phylogenetic reconstruction was performed using the Neighbor-Joining method in MEGA (version 11; Pennsylvania State University, University Park, PA, USA/Mega Limited, Auckland, New Zealand), generating trees based on the whole GyG1 genome and the VP1, VP2, and VP3 genes. Tree topology was validated by bootstrap analysis (1000 replicates) with absolute distances [26]. All eight GyG1 whole-genome sequences were deposited in GenBank. The metadata for these strains isolated from Guangxi and the 36 reference GyG1 strains are summarized in Table 3.

### 4.4. Recombination Analysis

Recombination events in the 8 Guangxi GyG1 strains and 36 reference strains were assessed using RDP (version 5.0; University of Oxford, Oxford, UK/University of Pretoria, Pretoria, South Africa) and SimPlot (version 3.5.1; Stuart Ray, Johns Hopkins University, Baltimore, MD, USA). Seven algorithms were used for detection, namely, RDP, GENECONV, BootScan, MaxChi, Chimaera, SiScan and 3Seq [27,28]. To balance the varying sensitivities and specificities of these integrated detection methods, a stringent criterion was adopted in this study: an event was only considered to be a confirmed recombination only if it was identified by at least five of the seven algorithms. This approach was implemented to minimize false positives that could arise from the potentially high false-positive rate of any single method.

## 5. Conclusions

In summary, on the basis of the results of the epidemiological and phylogenetic analyses in this study and considering the potential economic losses caused by GyG1 infection in the commercial chicken industry, carrying out more extensive investigations in live LPMs in China is necessary to clarify the geographical distribution characteristics of GyG1. The data obtained in this study can provide an important reference for the optimal design of GyG1 diagnostic methods and subsequent in-depth epidemiological studies in China.

## Figures and Tables

**Table 1:** Details of sampling from a live poultry market.

Year Market	Sample Type	A	B	C	D	E	F	G	H	Positive Rate	Total Positive Rate
2019	throat and cloacal swab	-	5/20 ^a^	4/20	-	-	-	-	-	9/40 ^a^ (22.50% ^b^)	9/48 (18.75%)
	environment sample *	-	0/4	0/4	-	-	-	-	-	0/8 (0.00%)	
2020	throat and cloacal swab	6/80	16/40	23/120	24/140	11/80	-	-	-	80/460 (17.39%)	94/552 (17.03%)
	environment sample	3/16	0/8	5/24	4/28	2/16	-	-	-	14/92 (15.22%)	
2021	throat and cloacal swab	26/108	20/128	19/104	20/148	29/144	6/40	-	-	120/672 (17.86%)	166/882 (19.67%)
	environment sample	12/40	3/40	10/34	14/48	5/40	2/8	-	-	46/210(21.90%)	
2022	throat and cloacal swab	14/100	16/100	18/60	28/120	26/120	-	-	-	102/500 (20.40%)	118/600 (19.67%)
	environment sample	0/20	1/24	0/12	5/20	10/24	-	-	-	16/100 (16.00%)	
2023	throat and cloacal swab	14/140	22/140	28/120	32/120	29/120	-	-	-	125/640 (19.53%)	146/888 (16.44%)
	environment sample	9/58	1/52	3/42	4/48	4/48	-	-	-	21/248 (8.47%)	
2024	throat and cloacal swab	1/40	6/60	1/20	9/40	0/20	0/20	4/60	3/121	24/381 (6.30%)	32/512 (6.25%)
	environment sample	1/8	1/18	0/4	2/8	0/4	3/18	1/30	2/49	8/131 (6.11%)	
Positive rate	throat and cloacal swab 460/2693(17.08%) **	61/468 (13.03%)	85/488 (17.42%)	93/444 (20.95%)	113/568 (19.89%)	95/484 (19.63%)	6/60 (10.00%)	4/60 (6.67%)	3/121 (2.48%)	565/3482 (16.23%)	
	environment sample 105/789(13.31%) ***	25/142 (17.61%)	6/146 (4.11%)	18/120 (15.00%)	29/152 (19.08%)	21/132 (15.91%)	3/18 (16.67%)	1/30 (3.33%)	2/49 (4.08%)		
Total positive rate		86/610 (14.10%)	91/634 (14.35%)	111/564 (19.68%)	142/720 (19.72%)	116/616 (18.83%)	9/78 (11.54%)	5/90 (5.56%)	5/170 (2.94%)		

^a^ Positive/total number of GyG1; ^b^ Positive rate of GyG1. * environment samples were collected from kitchen knives, chicken cages, bleeding buckets, chopping boards, floors, machinery, sewage, and tap water. ** The overall positivity rate for chicken swab samples; *** The overall positivity rate for environmental samples. A–H represent different live poultry markets.

**Table 2:** Recombination events involved in GX-AGV2-202109-5.

Event	Recombinant Strain	Major Parent	Similarity	Minor Parent	Similarity	*p*-Value						
						R	G	B	M	C	S	T
1	RS/BR/2015-Brazil	GX-AGV2-202109-5	96.90%	HN2019-H1	99.20%	ns	4.14 × 10^−2^	ns	3.93 × 10^−3^	2.63 × 10^−2^	1.26 × 10^−7^	3.07 × 10^−4^
2	HN2019-S1	JQ690763	98.2%	GX-AGV2-202109-5	99.4%	ns	7.79 × 10^−3^	8.09 × 10^−3^	6.41 × 10^−3^	8.19 × 10^−3^	1.62 × 10^−2^	3.31 × 10^−3^

Note: “ns” denotes not significant.

**Table 3:** Information about the sequences of the reference strains and the strains used in this study.

NO.	Strain Name	GenBank Accession	Year	Country	Host	Sample
1	Ave 3	HM590588	2008	Brazil	*Gallus gallus*	Serum
2	G17	KJ452213	2011	Hungary	*Ferret*	Feces
3	JQ690763	JQ690763	2012	China	*Homo sapiens*	Feces
4	S53/It	KU168250	2014	Italy	*Gallus gallus*	Serum
5	RS/BR/2015	KY039279	2015	Brazil	*Gallus gallus*	Feces
6	RS/BR/15/2S	MG846492	2015	Brazil	*Gallus gallus*	Feces
7	NX1506-1	KX708508	2015	China	*Gallus gallus*	Liver and spleen
8	NX1506-2	KX708509	2015	China	*Gallus gallus*	Liver and spleen
9	HLJ1506-1	KX708506	2015	China	*Gallus gallus*	Liver and spleen
10	HLJ1506-2	KX708522	2015	China	*Gallus gallus*	Liver and spleen
11	HLJ1510	KX708507	2015	China	*Gallus gallus*	Liver and spleen
12	JL1508	KX708511	2015	China	*Gallus gallus*	Liver and spleen
13	NX1510	KX708513	2015	China	*Gallus gallus*	Liver and spleen
14	JL1511	KX708516	2015	China	*Gallus gallus*	Liver and spleen
15	LN1511	KX708515	2015	China	*Gallus gallus*	Liver and spleen
16	HE1511	KX708514	2015	China	*Gallus gallus*	Liver and spleen
17	GS1512	KX708517	2015	China	*Gallus gallus*	Liver and spleen
18	GZ1601	KX708518	2016	China	*Gallus gallus*	Liver and spleen
19	JX1602	KX708519	2016	China	*Gallus gallus*	Liver and spleen
20	HLJ1603-1	KX708520	2016	China	*Gallus gallus*	Liver and spleen
21	HLJ1603-2	KX708521	2016	China	*Gallus gallus*	Liver and spleen
22	16CC1103	MK089245	2016	China	*Domestic cat*	Feces
23	17CC0315	MK089244	2017	China	*Domestic cat*	Feces
24	17CC0810	MK089246	2017	China	*Domestic cat*	Feces
25	HB2018-S1	MK840982	2018	China	*Snake*	Liver
26	AGV2-GXBS-26	OK245348	2019	China	*Dog*	Serum
27	AGV2-GXHG-32	OK245349	2019	China	*Dog*	Serum
28	HN2019-E1	OK540279	2019	China	*Egret*	whole blood
29	HN2019-S1	OK540280	2019	China	*Silver pheasant*	whole blood
30	HN2019-H1	OK540281	2019	China	*Hippopotamus*	whole blood
31	HN2019-T1	OK540282	2019	China	*Tiger*	whole blood
32	HN2019-PF1	OK540283	2019	China	*Peafowl*	whole blood
33	HN2019-SD1	OK540284	2019	China	*Sika deer*	whole blood
34	HN2019-L1	OK540285	2019	China	*Lion*	whole blood
35	HN2019-P1	OK540286	2019	China	*Common pheasants*	whole blood
36	GyVg1-SDAU-1	OL448986	2020	China	*Gallus gallus*	proventricular
37	AGV2-GX1901 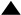	MW404233	2019	China	*Gallus gallus*	cloacal swab
38	AGV2-GX1902 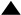	MW404234	2019	China	*Gallus gallus*	cloacal swab
39	AGV2-GX1909 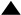	MW404235	2019	China	*Gallus gallus*	cloacal swab
40	AGV2-GX19010 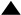	MW404236	2019	China	*Gallus gallus*	cloacal swab
41	AGV2-GX20-0918 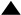	MW579760	2020	China	*Gallus gallus*	cloacal swab
42	AGV2-202109-5 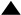	OP047911	2021	China	LPM	environmental sample
43	AGV2-202109-9 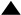	OP047912	2021	China	LPM	environmental sample
44	AGV2-20211-18 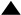	OP047913	2021	China	LPM	environmental sample

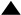
 The sequences obtained in this research.

## Data Availability

The original contributions presented in this study are included in the article and Supplementary Material. Further inquiries can be directed to the corresponding authors. The datasets generated and analysed during the current study are available from the corresponding author upon reasonable request.

## Supplementary Materials

The following supporting information can be downloaded at: https://www.mdpi.com/article/10.3390/ijms27041674/s1.
