# Supplementary material for: Molecular Characterization of Emerging Gyrovirus galga 1 from Poultry Markets of Guangxi, China
Source: Int J Mol Sci. 2026 Feb 9;27(4):1674. doi: 10.3390/ijms27041674 (PMC12940934; doi:10.3390/ijms27041674)
Supplement: Supplementary file 1 [file ijms-27-01674-s001.zip › ijms-4030906-supplementary.pdf]

Supporting Information

Molecular Characterization of Emerging *Gyrovirus galga* 1 from Poultry Markets of Guangxi, China

Table S1. Detect primer information.

Primer name	Sequence (5′→3′)	Product size/bp
GyG1-F	CGTGTCCGCCAGCAGAAACGAC	346
GyG1-R	GGTAGAAGCCAAAGCGTCCACGA	

The PCR was performed in a 25 μL reaction mixture consisting of 2 μL DNA template, 1 μL each of forward and reverse primers, 12 μL Premix Taq™ and 9 μL sterile distilled water. The thermal cycling protocol comprised an initial denaturation at 95 °C for 5 min; 34 cycles of 94 °C for 30 s, 58 °C for 30 s, and 72 °C for 30 s; and a final extension at 72 °C for 10 min. The amplified products were separated by electrophoresis on a 1.2% agarose gel.

Table S2. Primers used for PCR amplification of the GyG1 full genome.

Primer name	Sequence(5′→3′)	Position, nt
A1-F	CGTGGTAACCAACGATATGTAAGTA	
A1-R	CGTCGTTTGTGCTGACCATT	182-1393=1212
A1-FO	CGCTCCTCAACCTTTTCTTATTA	
A1-RO	GTACGCTGGGTCTTTTTGTCTC	126-1448=1323
A2-F	ACAATAAGCTTACCCTCTTTTTCC	
A2-R	AACGGACTTTCACCACGCAC	1161-2243=1083
A2-FO	GAGGGGTAAATTTCGATATGCG	
A2-RO	CTCGTTGTACCAGTTCACTTGC	1054-2305=1252
A3-F	GGATTACTGACGACACCATGGA	
A3-R	GGTATGTGCTCTGCTTATATCCCT	2076-435=736
A3-FO	TATTCAAAGGCTTGTTTCCCC	
A3-RO	CCGATACCAATTTGGATCTCTA	1901-704=1197
B1-F	GTCATCCTTATCTCCGACCAAT	
B1-R	GCGGTAGGGTCATCAGCTTC	268-1492=1225
B1-FO	CACAAAGATAACGCGAACCG	
B1-RO	ATTCTCTGGATTTAGTTGCAAGC	172-1576=1405
B2-F	ACTGCCACTAGACGCAAGGT	
B2-R	AATTTGTAGCTGTTTGCTCTGC	1251-2126=876
B2-FO	GACTGCGACGAAGACGCTATT	
B2-RO	CTTCTGTTGTTGCCGAGTGTC	974-2225=1252
B3-F	TCCTTCAACAAACATGTCATTAGAG	

B3-R	TTTAAGTTTCTTGCCCGTTGAT	1948-499=908
B3-FO	TAGACCCTGGTGAATACCTCCTT	
B3-RO	TCCCGGTTCCATTCAGGTAG	1799-533=1266

The PCR was conducted in a 50 μL reaction mixture consisting of 25 μL of 2× Gflex PCR Buffer, 1 μL of Tks Gflex™ DNA Polymerase, 1 μL each of forward and reverse primers, 4 μL of DNA template, and 18 μL of sterile distilled water. The thermal cycling protocol comprised an initial denaturation at 94 °C for 5 min; 34 cycles of 98 °C for 15 s, 56 °C for 15 s, and 68 °C for 45 s; and a final extension at 68 °C for 5 min. The amplified products were separated by electrophoresis on a 1.2% agarose gel.

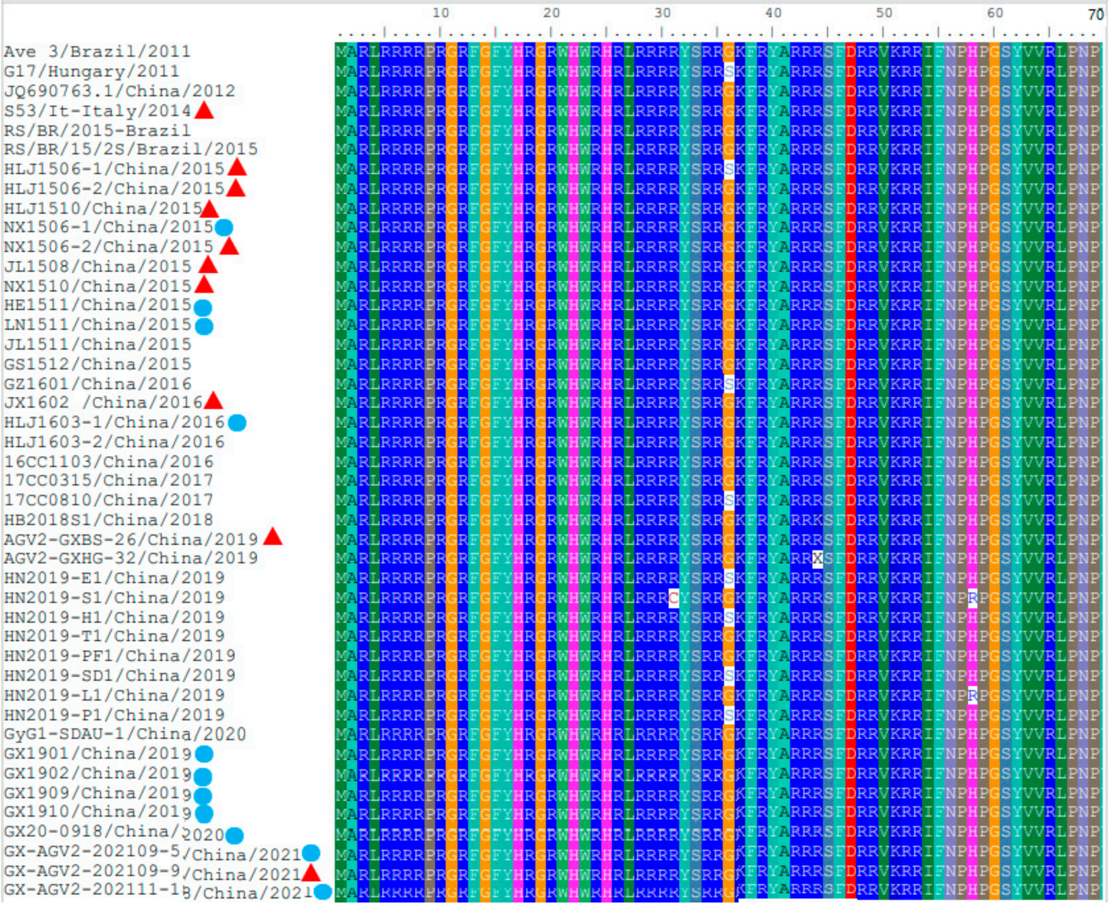

(A)

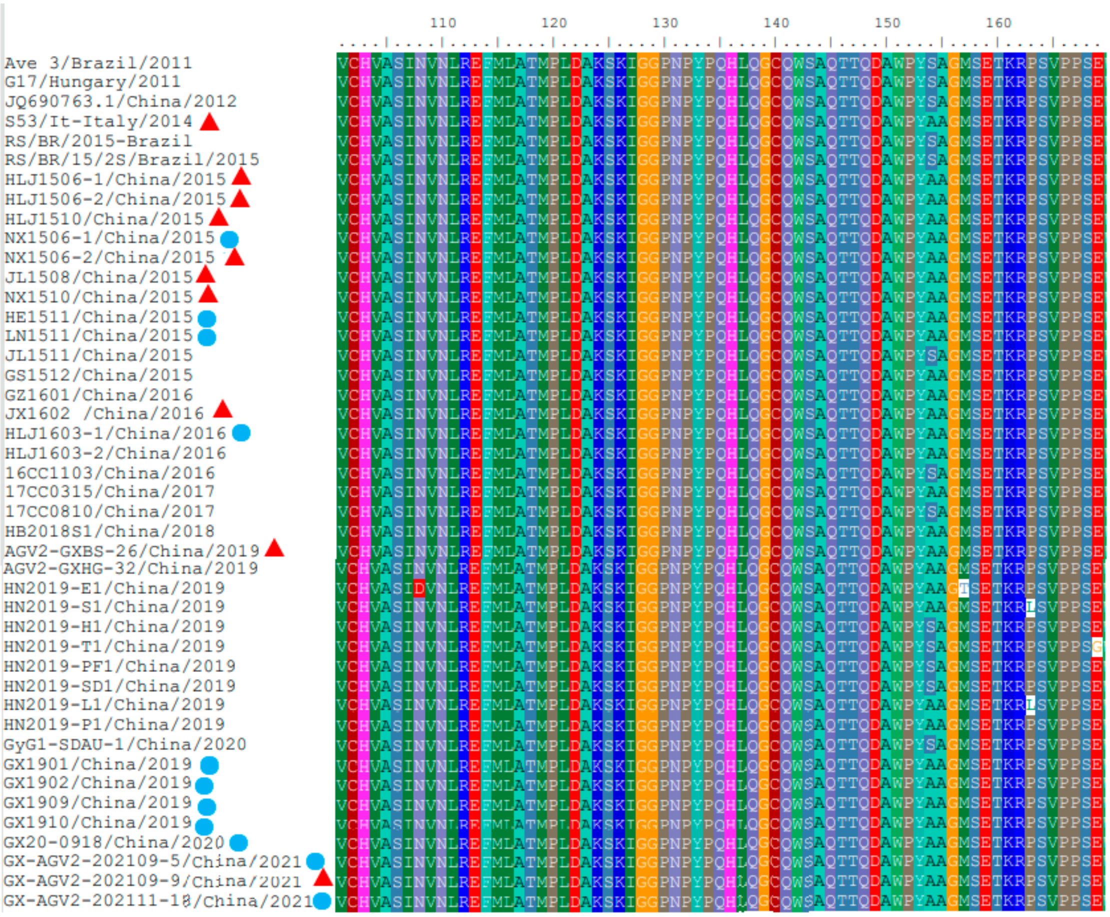
110
120
130
140
150
160
Ave 3/Brazil/2011
G17/Hungary/2011
JQ690763.1/China/2012
S53/It-Italy/2014
RS/BR/2015-Brazil
RS/BR/15/2S/Brazil/2015
HLJ1506-1/China/2015
HLJ1506-2/China/2015
HLJ1510/China/2015
NX1506-1/China/2015
NX1506-2/China/2015
JL1508/China/2015
NX1510/China/2015
HE1511/China/2015
LN1511/China/2015
JL1511/China/2015
GS1512/China/2015
GZ1601/China/2016
JX1602 /China/2016
HLJ1603-1/China/2016
HLJ1603-2/China/2016
16CC1103/China/2016
17CC0315/China/2017
17CC0810/China/2017
HB2018S1/China/2018
AGV2-GXBS-26/China/2019
AGV2-GXHG-32/China/2019
HN2019-E1/China/2019
HN2019-S1/China/2019
HN2019-H1/China/2019
HN2019-T1/China/2019
HN2019-PF1/China/2019
HN2019-SD1/China/2019
HN2019-L1/China/2019
HN2019-P1/China/2019
GyG1-SDAU-1/China/2020
GX1901/China/2019
GX1902/China/2019
GX1909/China/2019
GX1910/China/2019
GX20-0918/China/2020
GX-AGV2-202109-5/China/2021
GX-AGV2-202109-9/China/2021
GX-AGV2-202111-18/China/2021

(B)

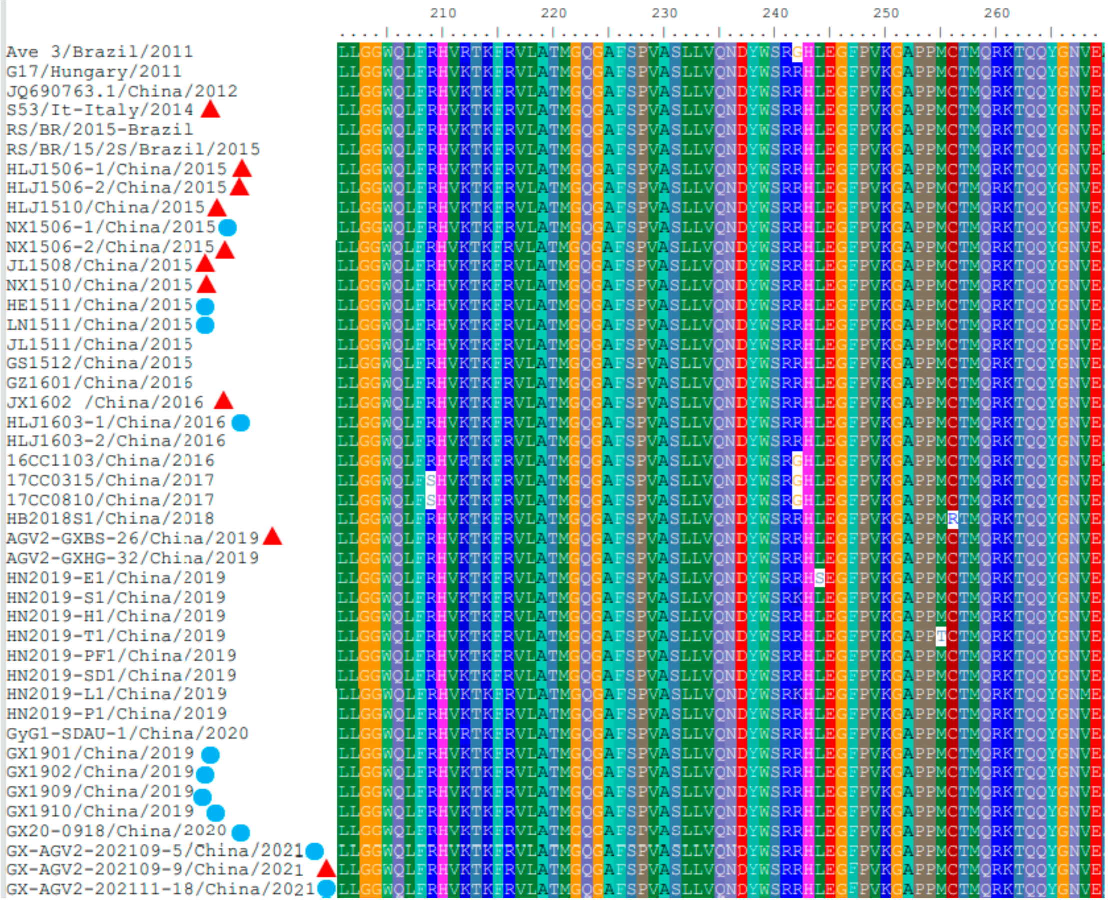
210
220
230
240
250
260
Ave 3/Brazil/2011
G17/Hungary/2011
JQ690763.1/China/2012
S53/It-Italy/2014
RS/BR/2015-Brazil
RS/BR/15/2S/Brazil/2015
HLJ1506-1/China/2015
HLJ1506-2/China/2015
HLJ1510/China/2015
NX1506-1/China/2015
NX1506-2/China/2015
JL1508/China/2015
NX1510/China/2015
HE1511/China/2015
LN1511/China/2015
JL1511/China/2015
GS1512/China/2015
GZ1601/China/2016
JX1602 /China/2016
HLJ1603-1/China/2016
HLJ1603-2/China/2016
16CC1103/China/2016
17CC0315/China/2017
17CC0810/China/2017
HB2018S1/China/2018
AGV2-GXBS-26/China/2019
AGV2-GXHG-32/China/2019
HN2019-E1/China/2019
HN2019-S1/China/2019
HN2019-H1/China/2019
HN2019-T1/China/2019
HN2019-PF1/China/2019
HN2019-SD1/China/2019
HN2019-L1/China/2019
HN2019-P1/China/2019
GyG1-SDAU-1/China/2020
GX1901/China/2019
GX1902/China/2019
GX1909/China/2019
GX1910/China/2019
GX20-0918/China/2020
GX-AGV2-202109-5/China/2021
GX-AGV2-202109-9/China/2021
GX-AGV2-202111-18/China/2021
(C)

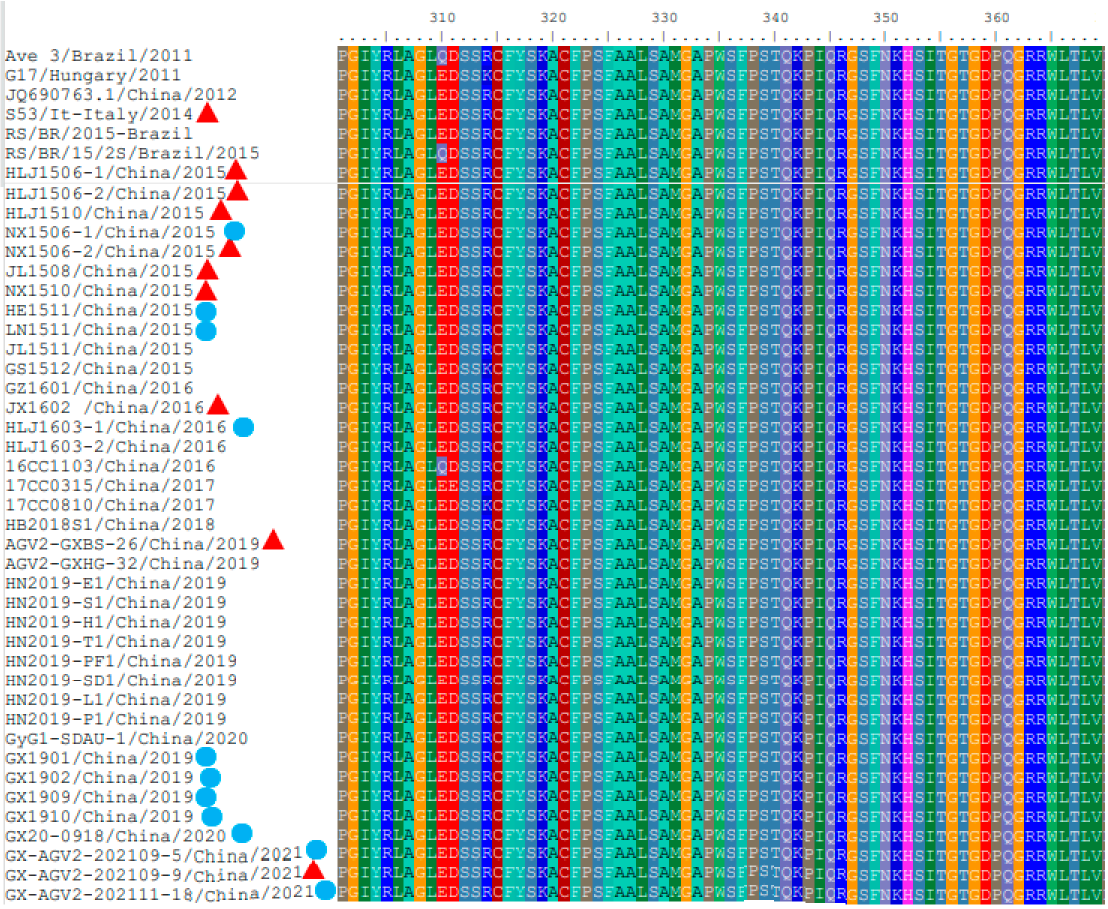
310
320
330
340
350
360
Ave 3/Brazil/2011
G17/Hungary/2011
JQ690763.1/China/2012
S53/It-Italy/2014
RS/BR/2015-Brazil
RS/BR/15/2S/Brazil/2015
HLJ1506-1/China/2015
HLJ1506-2/China/2015
HLJ1510/China/2015
NX1506-1/China/2015
NX1506-2/China/2015
JL1508/China/2015
NX1510/China/2015
HE1511/China/2015
LN1511/China/2015
JL1511/China/2015
GS1512/China/2015
GZ1601/China/2016
JX1602 /China/2016
HLJ1603-1/China/2016
HLJ1603-2/China/2016
16CC1103/China/2016
17CC0315/China/2017
17CC0810/China/2017
HB2018S1/China/2018
AGV2-GXBS-26/China/2019
AGV2-GXHG-32/China/2019
HN2019-E1/China/2019
HN2019-S1/China/2019
HN2019-H1/China/2019
HN2019-T1/China/2019
HN2019-PF1/China/2019
HN2019-SD1/China/2019
HN2019-L1/China/2019
HN2019-P1/China/2019
GyG1-SDAU-1/China/2020
GX1901/China/2019
GX1902/China/2019
GX1909/China/2019
GX1910/China/2019
GX20-0918/China/2020
GX-AGV2-202109-5/China/2021
GX-AGV2-202109-9/China/2021
GX-AGV2-202111-18/China/2021

(D)

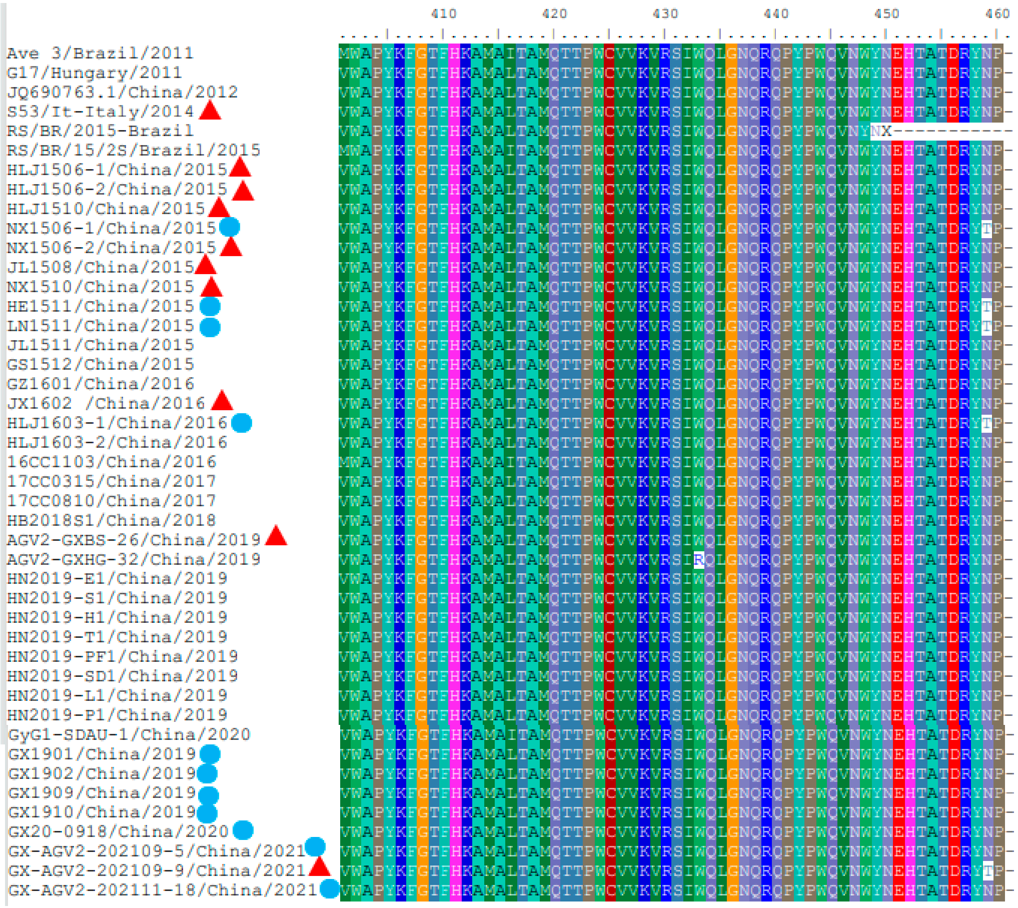

(E)

Figure S1: Sequence homology analysis revealed two groups with 100% VP1 amino acid identity (**A-E**). ▲The first group consisted of strain AGV2-202109-9 along with nine additional sequences S53/It, HLJ1506-1, HLJ1506-2, HLJ1510, NX1506-2, JL1508, NX1510, JX1602, and AGV2-GXBS-26VP1.●The second group contained seven sequences obtained in this study, together with the reference sequences NX1506-1, HE1511, LN1511, and HLJ1603-1.

.

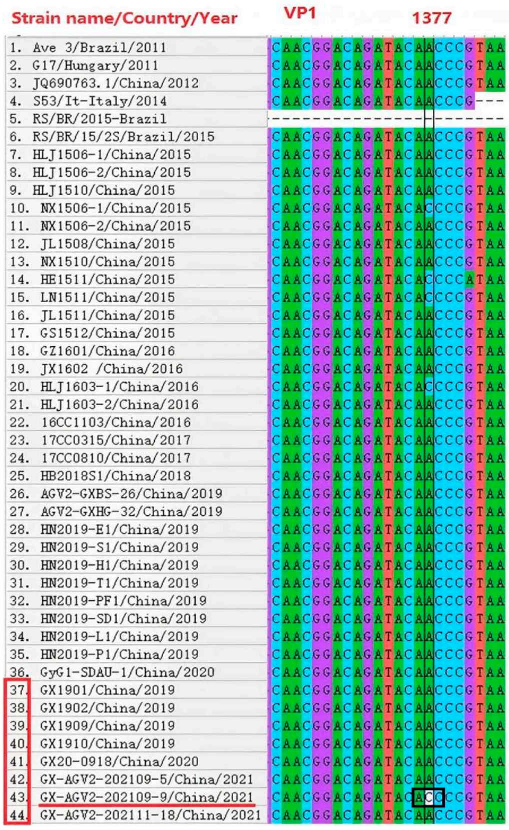

(A)

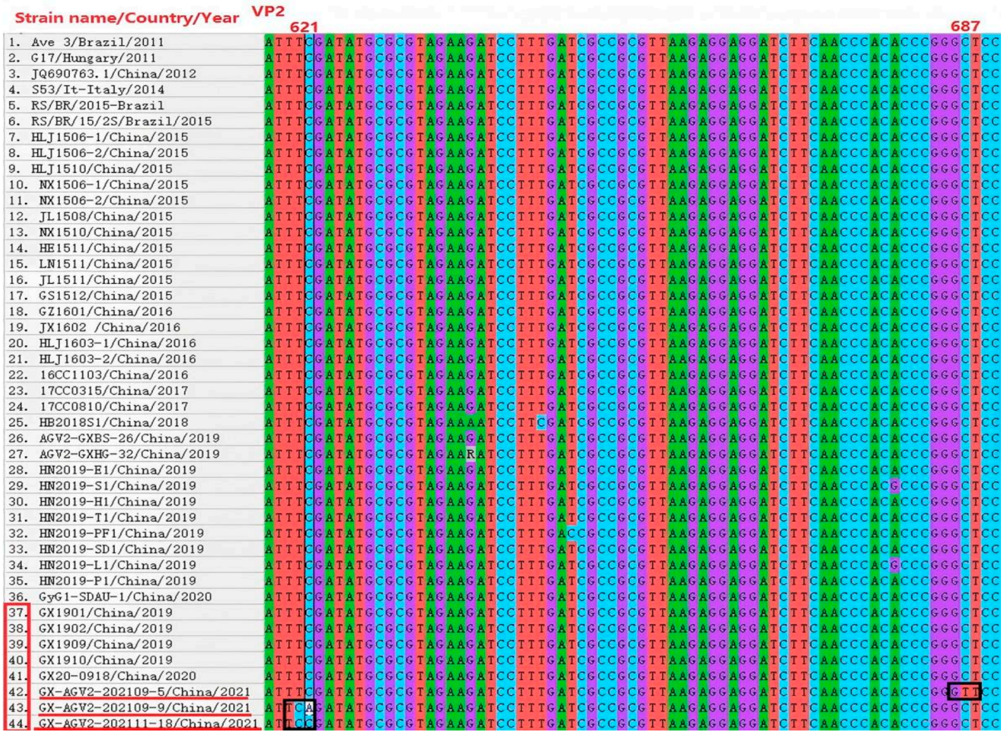

(B)

Figure S2: The nucleotide variations in VP1 and VP2 are shown in Figure S2A-2B. In the sequence alignment, the red box highlights the region corresponding to the strains analyzed in this study. Amino acid changes resulting from nucleotide mutations are indicated by black boxes, and any mutant strains are marked with red underscores.

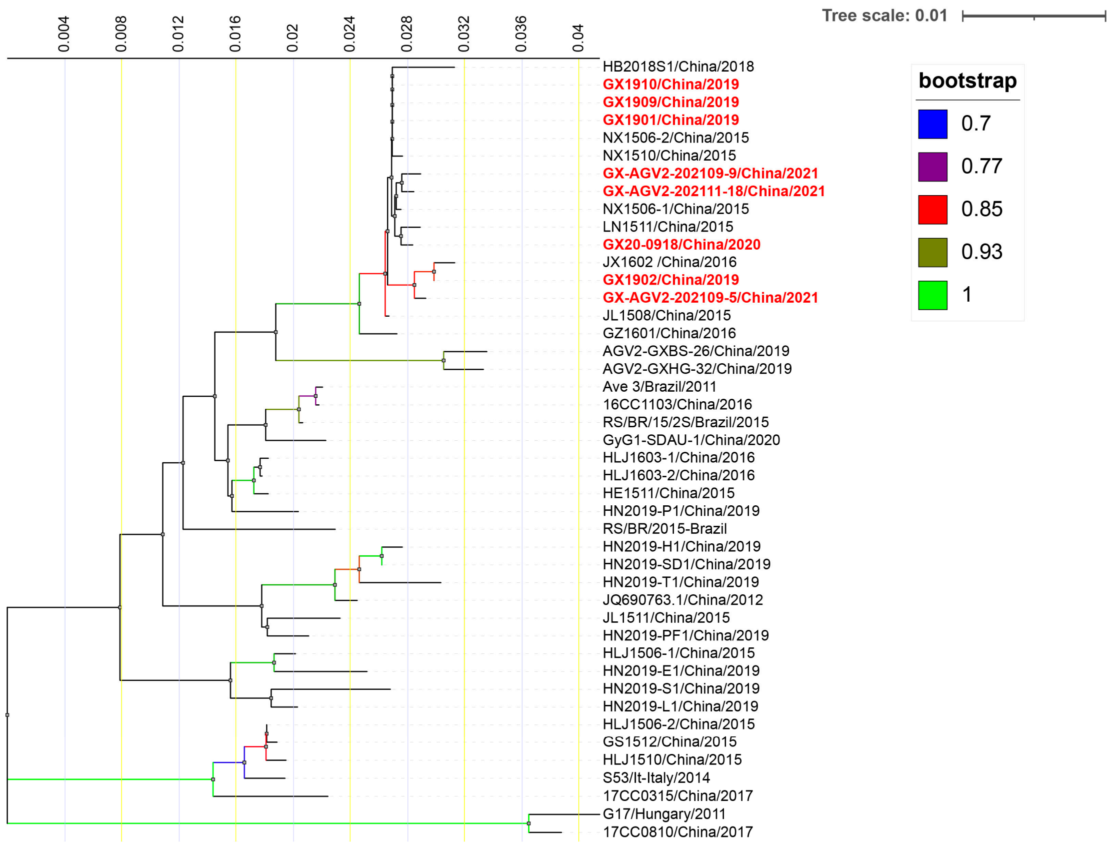
Tree scale: 0.01
0.004
0.008
0.012
0.016
0.02
0.024
0.028
0.032
0.036
0.04
HB2018S1/China/2018
GX1910/China/2019
GX1909/China/2019
GX1901/China/2019
NX1506-2/China/2015
NX1510/China/2015
GX-AGV2-202109-9/China/2021
GX-AGV2-202111-18/China/2021
NX1506-1/China/2015
LN1511/China/2015
GX20-0918/China/2020
JX1602 /China/2016
GX1902/China/2019
GX-AGV2-202109-5/China/2021
JL1508/China/2015
GZ1601/China/2016
AGV2-GXBS-26/China/2019
AGV2-GXHG-32/China/2019
Ave 3/Brazil/2011
16CC1103/China/2016
RS/BR/15/2S/Brazil/2015
GyG1-SDAU-1/China/2020
HLJ1603-1/China/2016
HLJ1603-2/China/2016
HE1511/China/2015
HN2019-P1/China/2019
RS/BR/2015-Brazil
HN2019-H1/China/2019
HN2019-SD1/China/2019
HN2019-T1/China/2019
JQ690763.1/China/2012
JL1511/China/2015
HN2019-PF1/China/2019
HLJ1506-1/China/2015
HN2019-E1/China/2019
HN2019-S1/China/2019
HN2019-L1/China/2019
HLJ1506-2/China/2015
GS1512/China/2015
HLJ1510/China/2015
S53/It-Italy/2014
17CC0315/China/2017
G17/Hungary/2011
17CC0810/China/2017
bootstrap
0.7
0.77
0.85
0.93
1

(A)

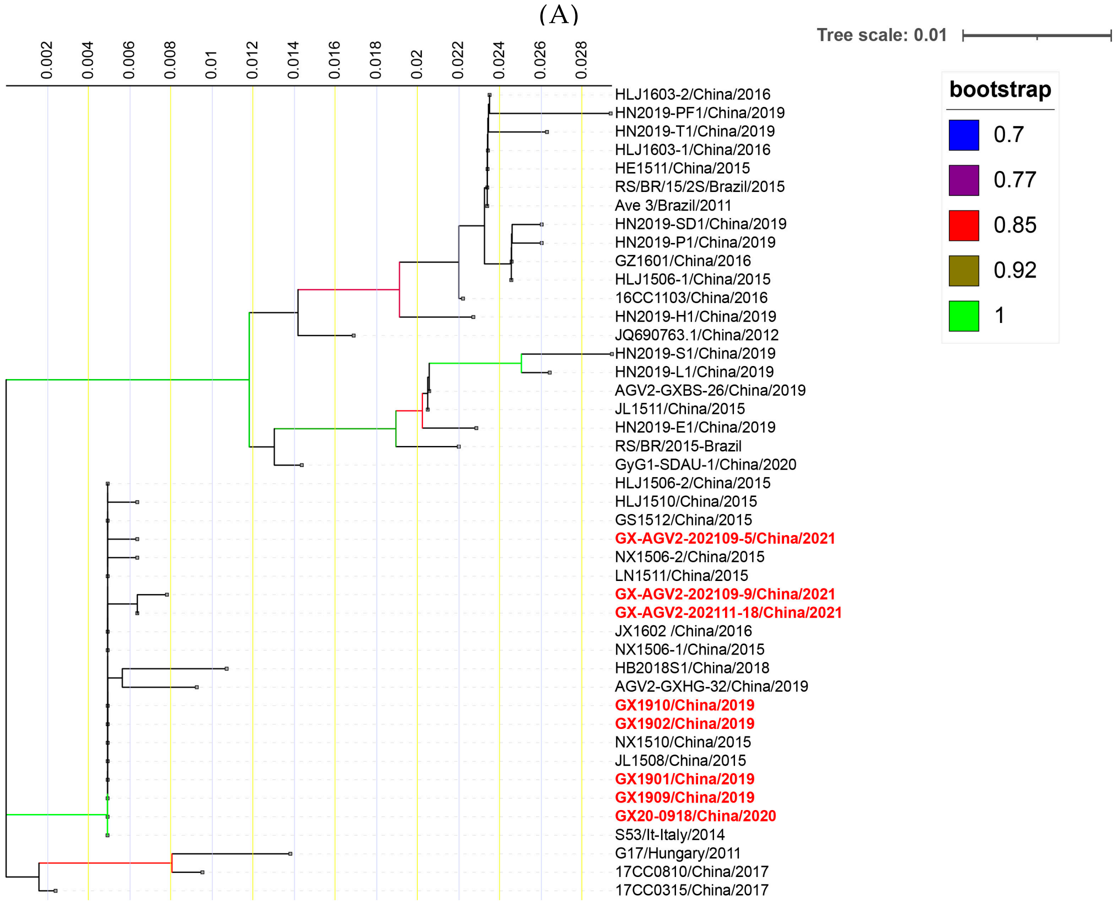
Tree scale: 0.01
0.002
0.004
0.006
0.008
0.01
0.012
0.014
0.016
0.018
0.02
0.022
0.024
0.026
0.028
HLJ1603-2/China/2016
HN2019-PF1/China/2019
HN2019-T1/China/2019
HLJ1603-1/China/2016
HE1511/China/2015
RS/BR/15/2S/Brazil/2015
Ave 3/Brazil/2011
HN2019-SD1/China/2019
HN2019-P1/China/2019
GZ1601/China/2016
HLJ1506-1/China/2015
16CC1103/China/2016
HN2019-H1/China/2019
JQ690763.1/China/2012
HN2019-S1/China/2019
HN2019-L1/China/2019
AGV2-GXBS-26/China/2019
JL1511/China/2015
HN2019-E1/China/2019
RS/BR/2015-Brazil
GyG1-SDAU-1/China/2020
HLJ1506-2/China/2015
HLJ1510/China/2015
GS1512/China/2015
GX-AGV2-202109-5/China/2021
NX1506-2/China/2015
LN1511/China/2015
GX-AGV2-202109-9/China/2021
GX-AGV2-202111-18/China/2021
JX1602 /China/2016
NX1506-1/China/2015
HB2018S1/China/2018
AGV2-GXHG-32/China/2019
GX1910/China/2019
GX1902/China/2019
NX1510/China/2015
JL1508/China/2015
GX1901/China/2019
GX1909/China/2019
GX20-0918/China/2020
S53/It-Italy/2014
G17/Hungary/2011
17CC0810/China/2017
17CC0315/China/2017
bootstrap
0.7
0.77
0.85
0.92
1

(B)

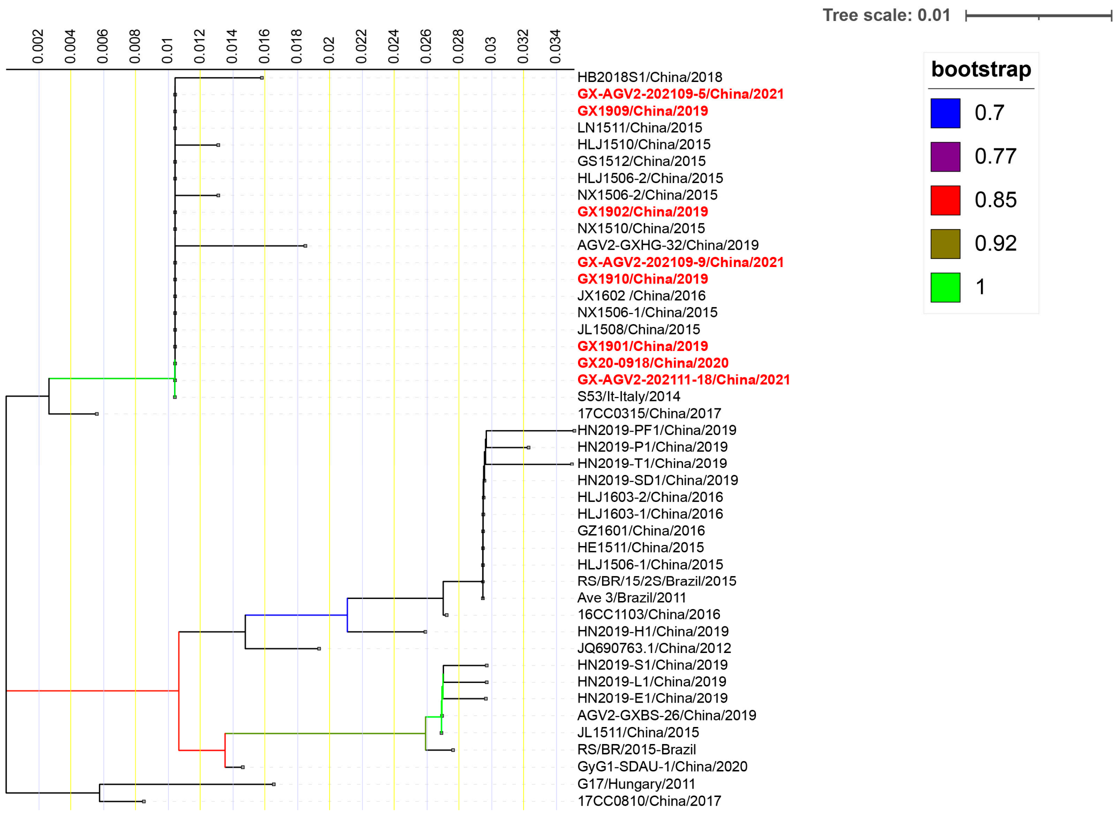

(C)

Figure S3: Phylogenetic trees were constructed using the nucleotide sequences of three homologous genome segments (VP1, VP2, and VP3)(**A-C**). The trees were generated with 1000 bootstrap replicates. The bar scale indicates genetic distance, and bootstrap values are displayed at the nodes. Red font represents the Guangxi GyG1 strains.
